# Profile of aeroallergen sensitizations in allergic patients living in southern Vietnam

**DOI:** 10.3389/falgy.2022.1058865

**Published:** 2023-01-04

**Authors:** Tu HK Trinh, Phuong TM Nguyen, Tai T Tran, Ruby Pawankar, Duy L Pham

**Affiliations:** ^1^ Center for Molecular Biomedicine, University of Medicine and Pharmacy at Ho Chi Minh City, Ho Chi Minh City, Vietnam; ^2^ Faculty of Medicine, University of Medicine and Pharmacy at Ho Chi Minh City, Ho Chi Minh City, Vietnam; ^3^ Unit of Allergy and Clinical Immunology, University Medical Center, Ho Chi Minh City, Vietnam; ^4^ Dept. of Pediatrics, Nippon Medical School, Tokyo, Japan

**Keywords:** allergens, house dust mite (HDM), cockroach, Vietnam, polysensitization

## Abstract

**Background:**

Climatic and geographical characteristics may alter the plant distribution and thereby the patterns of allergens.

**Objective:**

To evaluate the profile of allergen sensitization in patients in southern Vietnam and its association with allergic diseases.

**Methods:**

We collected data of 423 patients who visited the Unit of Allergy and Clinical Immunology, University Medical Center, Vietnam from 2014 to 2021, from their medical records. Patients underwent skin prick tests to the 12 most common aeroallergens. Clinical evaluation and diagnosis of allergic diseases was done in consert with their allergen sensitization status.

**Results:**

Mites and cockroach were the most prevalent sensitizing allergens, with the sensitization prevalences as followed: *Dermatophagoides farinae* (Df) (59.8%), *Dermatophagoides pteronyssinus* (Dp) (50.4%), *Blomica tropicalis* (Bt) (49.6%), storage mites mix (10.4%), and cockroach (10.2%). Sensitization to Df was more predominant in males than in females (66% *vs* 54.1%). Dp-sensitized patients were younger than non-sensitized patients (29.01 ± 13.60 *vs.* 32.17 ± 14.89) whereas storage mites-sensitized patients were older than the non-sensitized groups (36.25 ± 13.53 *vs.* 28.76 ± 13.39) (*p *< 0.05 for all). A considerable proportion of patients with urticaria, allergic rhinitis, and atopic dermatitis were sensitized to mites. Polysensitization to different species of house dust mites (Dp, Df) and storage mites (Bt) was prevalent among patients sensitized to any kind of mites.

**Conclusions:**

Among people living in southern Vietnam, HDM mites, and cockroach were the predominant allergens. Further studies on the factors regulating the association between allergen sensitization with allergic diseases and polysensitization are crucial.

## Introduction

The prevalence of allergic diseases is increasing worldwide, in both developed and developing countries. Various clinical manifestations of allergic diseases have been documented, including asthma, allergic rhinitis (AR), anaphylaxis, allergies to drugs, food and insect venom, atopic dermatitis (AD), urticaria, and angioedema. According to the World Health Organization, hundreds of millions of people suffer from rhinitis, while 300 million subjects affected by asthma. The high prevalence of allergic diseases has a negative impact on the patients' quality of life and also the socio-economics and welfare (1). In this context several researchers have investigated the patterns and characteristics of allergens in association with these allergic diseases. According to the World Allergy Organization, sensitization to airborne particles occurs in 40% of the population and is strongly associated with exposure to pollens, molds, dust mite and to cockroach (2). It was demonstrated that sensitization to any allergens is a risk factor for the development of asthma and eczema, however, the implicated allergens varied (3). In the United States of America, studies using prick-puncture allergy skin tests demonstrated that 54.3% of participants displayed positive reactions to at least 1 type of allergen, with house dust mite (HDM) ranked first (27.5%), followed by perennial rye (26.9%), short ragweed (26.2%), German cockroach (26.1%), and others (4). In addition to HDM, a major allergenic source, yet there are few reports about the association of molds and pollens to asthma and pollinosis, respectively (5–7). Interestingly, the distribution of allergens can vary between regions depending on environmental factors, such as geography and climatic factors (8). In the southern China, less than 10% were sensitized to pollen and molds, while more than 20% of subjects in central and eastern parts were sensisitzed to pollen and mold (9).

In Vietnam, the diverse topography and climatic conditions divide the whole country into multiple climatic regions. Therefore, characteristics of flora, fauna, and mold may vary, which leads to differences in susceptible allergens among different locals. In a study conducted in the northern part, the researchers demonstrated that 33.8% of allergic patients had positive skin prick tests (SPTs) to at least 1 type of aeroallergens, despite gender differences, with the highest sensitization rate being to HDM and cockroach (10).

The predominance of HDM/cockroach-sensitization was observed in Vietnam's Central Highland and Central Coast (11, 12). However, there is a lack of studies conducted in southern Vietnam. Given the variable geographic and climate changes between regions, we performed this study to investigate the allergen sensitization profile of patients living in the southern Vietnam. Furthermore, we aimed to elucidate the association between allergen sensitizations and allergic diseases in comparison with other areas in Vietnam.

## Materials & methods

### Study population and SPT

In this study, we enrolled all Vietnamese patients who were living in Vietnam's southern provinces and visited the Unit of Allergy and Clinical Immunology, University Medical Center, Ho Chi Minh City, Vietnam from 2014. All patients underwent SPT to standardized extracts from 12 aeroallergens [*Dermatophagoides farinae* (Df*), Dermatophagoides pteronyssinus* (Dp), *Blomica tropicalis* (Bt), German cockroach, cat hair, dog hair, mouse epithelia, Bermuda grass, storage mites mix, *Alternaria* **spp*, Cladosporium *spp,** and *Aspergillus* spp (Starllergenes Greer, London, United Kingdom)]. The inclusion criteria included patients who had at least a positive SPT to at least one aeroallergen. This study was approved by the Ethics Committee of the University of Medicine and Pharmacy at Ho Chi Minh City.

### Study design and data collection

We performed a cross-sectional study using the medical records collected at the Unit of Allergy and Clinical Immunology. Patients' demographic data, physician-diagnosed allergic diseases, including asthma, AR, allergic conjunctivitis, AD, urticaria, etc, and SPT results of tested aeroallergens were collected. For every participating subject, the diagnosis was established, and treatment was administered according to the approved guidelines of the University Medical Center. Polysensitization is defined as sensitization to at least two allergens (13).

### Statistical analysis

The normality test of data was checked with the Kolmogorov-Smirnov test. For continuous variables, means were calculated and compared between the 2 groups by the Student's *t*-test or Mann Whitney *U* test, depending on the normality of data. For categorical variables, data were coded for analysis and calculated as a percentage. Comparisons between groups were made by Pearson's chi-square test or Fisher's exact test. Data input and graph preparation was conducted using Microsoft Excel (Microsoft Corporation, Washington, United States). All analysis was performed by using statistic software packages IBM SPSS 20.0 (Armonk, NY, United States), with a significance level at *p *< 0.05. Venn diagrams were prepared by jvenn (14).

## Results

### Characteristics of the study population

In this study, 423 patients were evaluated. The demographic characteristics are as shown in Table 1. There were 198 males (46.8%) *vs.* 225 females (53.2%), with a male:female ratio of 0.88. The mean age of the study population was 30.71 ± 14.22 years (range: 5–84 years old). Children accounted for 79 patients (18.7%), while 344 patients (81.3%) were adults. Regarding the area of residence, 259 patients (69.7%) lived in the urban area, which was predominant compared to 128 (30.3%) patients who were living in the rural area.

**Table 1:** Demographic characteristics of study subjects.

Characteristics	Total (*n*, %)
Age (years)	30.71 ± 14.22
Children	79 (18.7)
Adult	344 (81.3)
Gender	
Male	198 (46.8)
Female	225 (53.2)
Place of residence	
Urban	295 (69.7)
Rural	128 (30.3)

Data were shown as means ± SD for continuous variables and % for categorical variables.

### Prevalence of positive SPT in the study population

**Figure 1:**
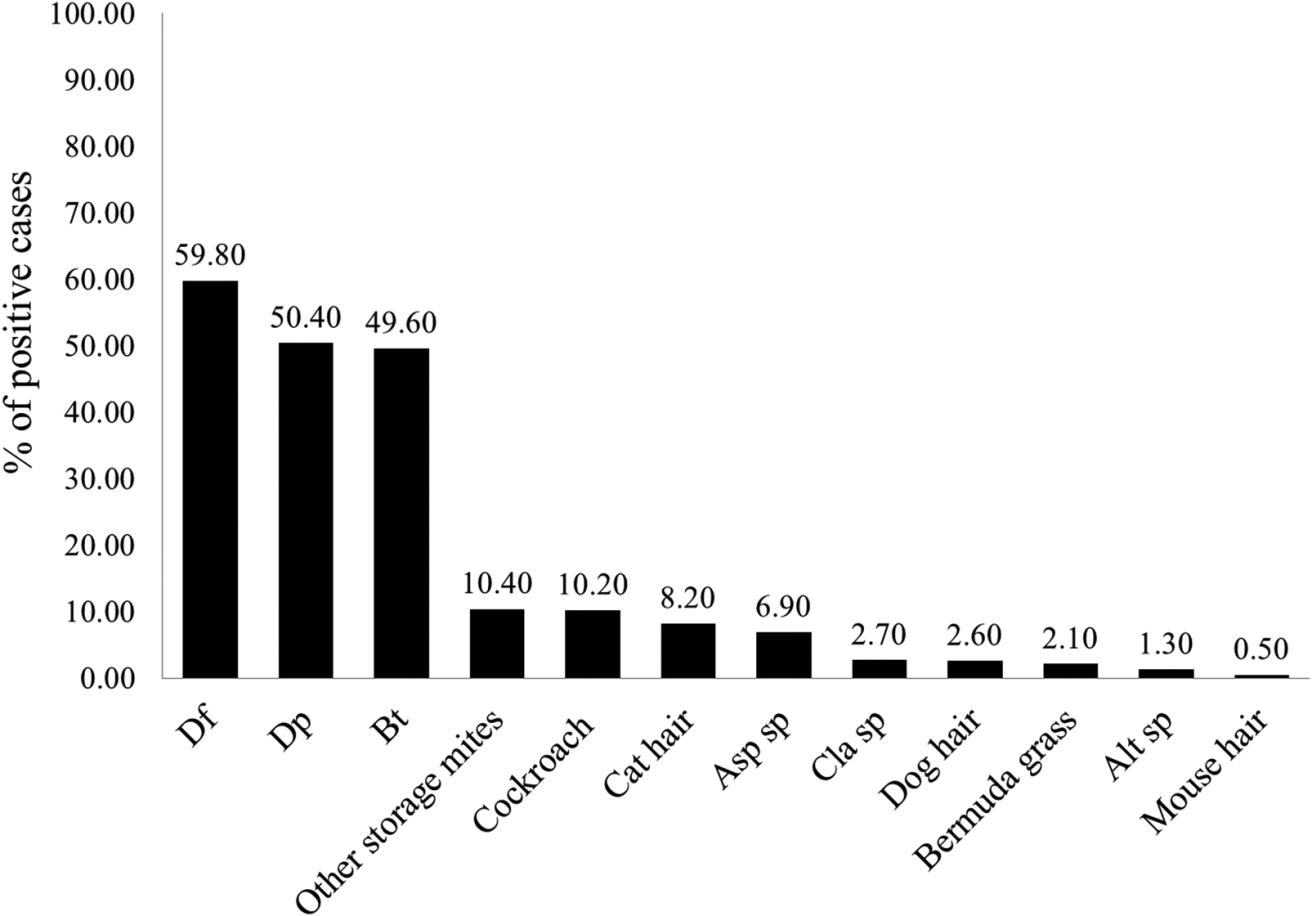
The prevalence of positive responses in SPTs to aeroallergens. Data were presented as percentage. The ratio of patients with positive SPT (as defined in the Materials & Methods section) in the study population was calculated.

As shown in Figure 1, the highest rate of positive SPT was noted for Df (59.8%), followed by Dp (50.4%), Bt (49.6%), storage mites (10.4%) and cockroach (10.2%). Regarding animal allergens, the positive rates of cat hair and dog hair were 8.2% and 2.6%, respectively. In terms of molds, the positive rates for Asp, Cla và Alt were found to be 6.9%; 2.7% and 1.3%, respectively.

### Demographic characteristics of subjects with positive SPT

It was found that male subjects were more likely to have positive SPT to Df than females (66.0% *vs.* 54.1%, *P *= 0.015). Subjects with positive SPT to Dp were significantly younger as compared to subjects with negative SPT (29.01 ± 13.60 *vs.* 32.17 ± 14.89; *p *= 0.027). In contrast, subjects with positive SPT to storage mites were older than subjects with a negative SPT (36.25 ± 13.53 *vs.* 28.76 ± 13.39; *p *= 0.019). There were no significant differences in allergen sensitization among patients living in urban and rural areas (Table 2).

**Table 2:** Dermographic chracteristics of subjects with postive SPT, according to allergens.

Allergens	Age (years)			Gender* (*n*, %)			Living area* (*n*, %)		
	Postive	Negative	*P*	Male (*n* = 198)	Female (*n* = 225)	*P*	Urban (*n* = 295)	Rural (*n* = 128)	*P*
Df (*n* = 400)	30.68 ± 14.47	30.26 ± 14.05	0.773	126 (66.0)	113 (54.1)	0.015	167 (59.4)	72 (60.5)	0.841
Dp (*n* = 401)	29.01 ± 13.60	32.17 ± 14.89	0.027	100 (52.4)	102 (48.6)	0.449	143 (50.9)	59 (49.2)	0.752
Bt (*n* = 401)	30.04 ± 13.28	31.11 ± 15.30	0.456	93 (48.7)	106 (50.5)	0.721	144 (51.2)	55 (45.8)	0.321
Storage mites (*n* = 193)	36.25 ± 13.53	28.76 ± 13.39	0.019	12 (11.0)	8 (9.5)	0.737	13 (9.6)	7 (12.1)	0.610
Cockroach (*n* = 196)	34.80 ± 14.69	28.76 ± 13.20	0.057	14 (12.7)	6 (7.0)	0.187	13 (9.4)	7 (12.3)	0.539

Bt, blomia tropicalis; Df, dermatophagoides farinae; Dp, dermatophagoides pteronyssinus.

Continous data were shown as mean ± SD, and *P* values were analyzed by Student's *t* test.

* Data were described as n (%). *P* values were calculated by Pearson's Chi square.

### Association between sensitization status and allergic diseases among the study population

Urticaria, AR and AD were the most prevalent allergic diseases recorded in this study population, with the prevalence as 32.9%, 18.2%, and 5.2%, respectively. Therefore, we selected these 3 diseases to investigate the relationship between allergen sensitization and allergic diseases. Patients who were diagnosed with a single allergic disease were included to avoid misinterpretation of the data due to the impact of comorbid allergic diseases. Most of the patients were found to have positive SPT to 3 HDM species (40.7–59.3%) (Table 3). Firstly, patients with urticaria were highly associated with Df, Dp, and Bt, with a positive prevalence of 59.3%, 47.2%, and 40.7%, respectively. Secondly, among patients with AD, we noted the rates of sensitization to Dp, Df, and Bt as 59.2%, 42.9%, and 42.9%, respectively. Regarding AR, we observed the high prevalence of sensitization to mites, as followed by Df (50.7%), Dp (50.7%) and Bt (57.7%). However, no significant differences in allergen sensitization among the 3 disease groups were found. Sensitization to storage mites and cockroach were observed only in patients with AR, but not in those with urticaria or AD. Interestingly, the polysensitization rate was significantly higher among patients with AR compared to patients with urticaria (*p *= 0.047).

**Table 3:** Profile of allergens screened in patients with allergic diseases Fisher's exact tests.

Sensitized allergens (*n*, %)	Urticaria (*n* = 123)	AR (*n* = 71)	AD (*n* = 49)	*P*		
				Urticaria vs. AR	Urticaria vs. AD	AR vs. AD
Df	73 (59.3)	36 (50.4)	29 (59.2)	0.055	0.986	0.128
Dp	58 (47.2)	36 (50.7)	21 (42.9)	0.838	0.611	0.756
Bt	50 (40.7)	41 (57.7)	21 (42.9)	0.095	0.763	0.295
Storage mites	0 (0.0)	8 (11.3)	–	0.378	–	–
Cockroach	0 (0.0)	10 (14.1)	–	0.317	–	–
Polysensitization	63 (51.22%)	47 (66.12%)	21 (42.86%)	0.047	0.550	0.405

AR, allergic rhintis; AD, atopic dermatitis; Bt, blomia tropicalis; Df, dermatophagoides farinae; Dp, dermatophagoides pteronyssinus. Data were described as *n* (%). *P* values were calculated by Pearson's Chi square or Fisher.

### Polysensitizations to mites and other allergens

There were 377 patients (94%) who showed positive results to at least 1 of the mites (Df, Dp or Bt). Regarding the concomitant positive SPT responses to 2 mites, there were 124 patients (31.5%) who were positive to Df and Dp, 102 patients (25.7%) who were positive to Df and Bt, and 106 patients (26.7%) who were positive to Dp and Bt. Seventy-two patients (18.2%) were sensitized to all 3 mites (Table 4).

**Table 4:** Polysensitization to HDM.

SPT positive to	Percentage *n*, (%) (*n* = 401)
At least 1 HDM	377 (94.01)
Df and Dp	124 (31.5)
Df and Bt	102 (25.7)
Dp and Bt	106 (26.7)
Df, Dp and Bt	72 (18.0)

Bt, blomia tropicalis; Df, dermatophagoides farinae; Dp, dermatophagoides pteronyssinus. Data were shown as *n* (%).

**Figure 2:**
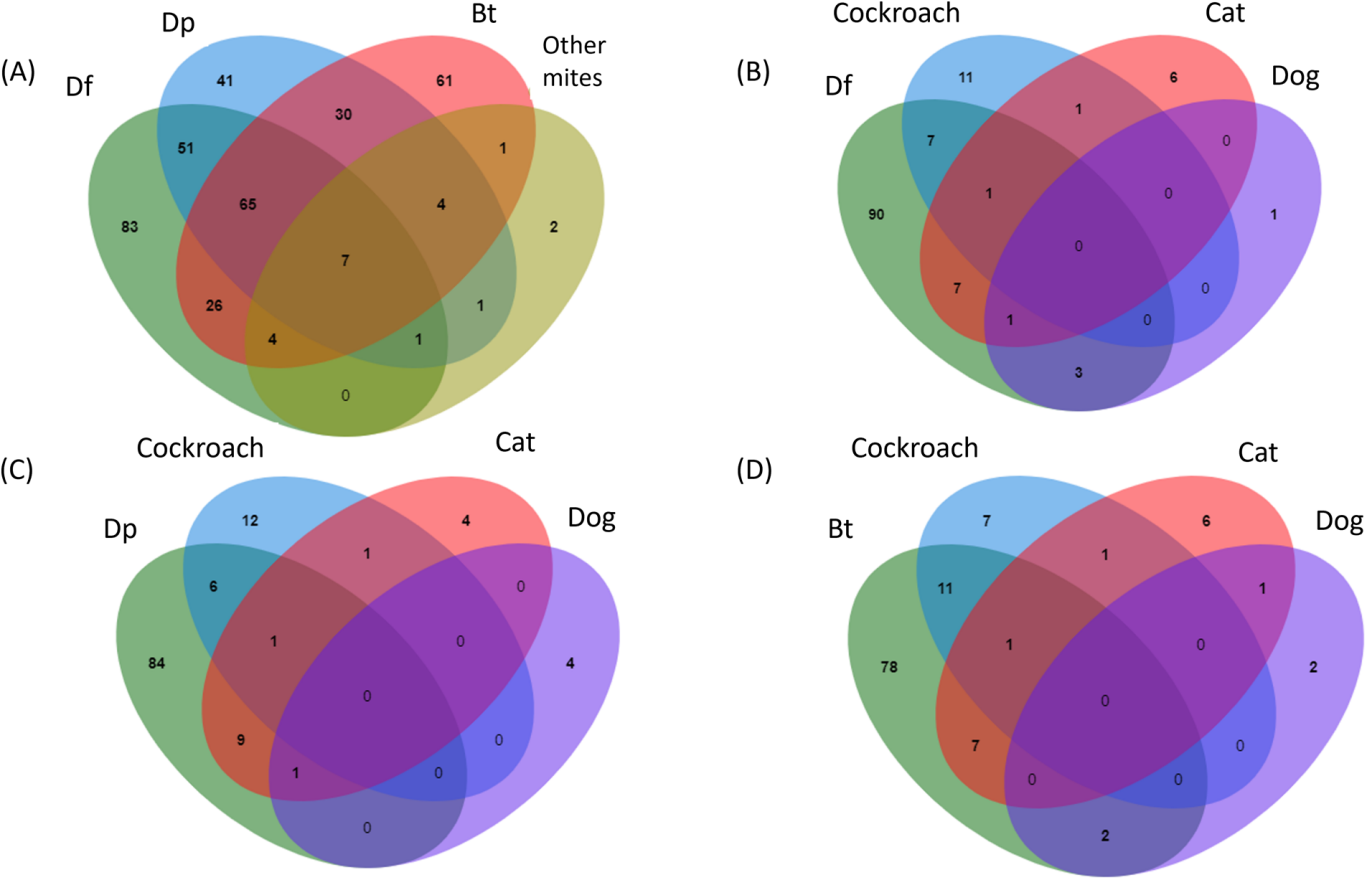
Patterns of polysensitization between the inhalant allergens. Venn diagrams showed the (**A**) polysensitization between Dp, Df, Bt and storage mites, and polysensitization between Dp (**B**), Df (**C**), and Bt (**D**) with extracts from cockroach, cat and dog hair. Bt, Blomia tropicalis; Df, Dermatophagoides farinae; Dp, Dermatophagoides pteronyssinus.

In Figure 2, polysensitizations to mites and other allergens were extensively investigated. Among Df, Dp, Bt and storage mites, 7/364 (1.92%) subjects were polysensitized to Df, Dp, Bt and storage mites (Figure 2A). Compared to other indoor allergens such as cockroach, cat hair and dog hair (Figures 2B–D), mite-sensitized subjects were more frequently simultaneously sensitized to cockroach and cat hair than dog hair. The prevalences of cockroach and Df/Dp/Bt co-sensitized subjects were 8/194 (4.12%), 7/194 (3.61%), 12/194 (6.19%), respectively. Meanwhile, the prevalences of cat hair and Df/Dp/Bt co-sensitized subjects were 9/194 (4.64%), 11/194 (5.67%), 8/194 (4.12%), respectively. Fungi, mouse hair and Bermuda grass were not included for analysis of co-sensitization due to the low positivity rate.

## Discussion

In this study, we investigated the profile of aeroallergen sensitizations of 423 Vietnamese patients with allergic diseases, who were living in the southern part of Vietnam. Mites and cockroach were the most prominent allergens, with sensitization to mites was found mostly in patients with allergic diseases (urticaria, AD and AR). The polysensitization to different HDM species (Dp, Df, Bt, and storage mites) was relatively high among the tested subjects.

Various studies in Southeast Asia have reoprted that mites and cockroach are the highly sensitizing aeroallergens, in both children and adults. A study from Thailand reported three popular allergens, including mixed mites (62.2%), mixed cockroaches (61.1%) and HDM (48.9%) (15). In Korea, children with AR were mostly sensitized to mites (87.3%) (16). The reports from Vietnam, including our study, were in line with these results (7, 10, 17). The high prevalence of sensitization to mites and cockroach from all over Vietnam can be attributed to the monsoon tropical climate, which is a favorable environment for the development and reproduction of these species (18). However, studies from other parts of Vietnam revealed different patterns of popular mites. The present study showed the high prevalence of sensitization against Df, followed by Dp, Bt, storage mites and cockroach. Among patients with chronic respiratory diseases in southern Vietnam, Df, Bt, Dp and cockroach droppings were the most frequent sensitizers (17, 19). Another study from northern Vietnam showed the high prevalence of sensitization to Bt, Dp, Df, and cockroach, while in the Central Highlands region, Dp was the predominant allergen, followed by Df, Bt, and cockroach (7, 10, 12). These differences may be interpreted by the fact that Vietnam spans over a wide range of latitude with diverse topographical features causing variation of climate, humidity, and temperature characteristics. Moreover, mites and cockroach can adapt to different environments and climates. Thus, the high prevalence of sensitization to these species was observed in not only tropical but also temperate climates.

Gender, age, and living area were determined as independent predictors of 1 or more positive SPT responses (4). We analyzed the patterns of sensitization by stratification of gender, age, and living area. A male predominance in sensitization prevalence to Df was observed in the present study, which was also reported in a study conducted in Vietnamese people living in the northern part of Vietnam (7). In comparison to worldwide data, National Health and Nutrition Examination Survey (NHANES) III advocated for the sex-specific differences in HDM sensitization rates with male predominance (4). A systematic review additionally highlighted that the prevalence of Df sensitization was more pronounced in men (20). A higher male:female ratio of sensitization to mites may be due to different working environments or sex hormones. Men are more likely to endure heavier labor and are more exposed to dust than women, thus, they seem to have a higher sensitization to HDM. Several hypotheses regarding the effects of sex hormones on immune responses have been suggested (20). Further studies on the associations between HDM sensitization and genders are needed.

Subsequently, we analyzed the association of sensitization prevalence with age and place of residence. Dp-sensitized patients were younger compared to non-sensitized subjects. In northern Vietnam, the prevalence of mites sensitization (Dp, Df, Bt) was more common in patients less than 45 years old comparing their older counterparts (7). NHANES II reported that the sensitization prevalence to at least 1 allergen was highest in patients aged 12–24 years old, and dropped in patients aged 65–74 years old (21). The decline of sensitization prevalence with age may be explained by the immunosenescence in both humoral and innate immunity, leading to the lowered responses of total and specific IgE (22–24). Moreover, long-term exposure to antigens may induce tolerance, which results in higher Dp-sensitized rates in younger patients than in the older patients (25). Notably, we are the first group in Vietnam to report that storage mites-sensitized patients were older than non-sensitized ones. Storage mites can be found living in agricultural products such as rice grains, wheat, peanut, and straw, which were found in the working environment where adults are more likely to work in and exposed to this type of mites than children (26, 27). Nevertheless, we could not observe any significant differences in terms of the living area. This is in contrast with a study showing that being born in an urban area was a risk factor for sensitization to mites (17). The disparity among studies in a similar geographical region may be due to different study populations. All patients with different patterns of allergic status were recruited in the present study, while the other study preferentially selected patients with chronic respiratory diseases. In general, among different areas of Vietnam, age-dependent sensitization to mites was replicated, but the effects of the census region on allergen sensitization need to be carefully interpreted.

Next, we aimed to see associations between airborne allergens with various allergic diseases. Overall, we observed a high prevalence of mite sensitization with urticaria, AR and AD. The prevalences of mite-sensitization in patients with urticaria and AD have fluctuated between studies, in which, several studies reported similar prevalence to our findings (28–31). For instance, studies from India and the Czech Republic reported the prevalence of mites-sensitized patients with urticaria and AD as 53.0% and 61.0%, respectively (30). Regarding AR, in northern Vietnam, a strong association between mite sensitization and AR was found, however, the prevalence was slightly lower than in our study, with 28.1%–30.9% of patients with AR symptoms (7). The gap between these studies was assumed to be due to different diagnostic methods. We collected data from medical records, meanwhile in the previous study, information on diseases was obtained from a community-based survey. Therefore, we will need to confirm these findings in studies at national levels with a unified method.

Polysensitization is important upon evaluating allergen profiles, given that polysensitization tends to be associated with more severe clinical outcomes (32). In our study, most of the patients were polysensitized to 2 or 3 mites (Dp, Df, and Bt). Similar studies advocated for the polysensitization between Dp, Df and Bt (7, 33). Indeed, the high polysensitization prevalence to mites was widely accepted, for instance, in the United States, 34% of patients with asthma/AR were sensitized to 3 mites (Df, Dp, Bt), while only 7% of patients were monosensitized (34). That could be resulted from the similarity in epitopes of mites, leading to cross-reactivity between mites-specific IgE (35, 36). Additionally, although there is evidence regarding the cross-reactivity between dog and cat hair, the polysensitization in our study was low (37). Thus, we need further studies evaluating the polysensitization and its association with disease severity in the Vietnamese cohort.

We conducted a retrospective, cross-sectional study design based on medical records in one single center. However, the data are limited, and we could not observe the longitudinal changes of sensitization as well as to follow-up patients with allergic diseases. Secondly, the profile of pollen sensitization was not included in the study, partly due to the lack of commercial allergen extracts from indigenous plant species. Thus, it is necessary to develop relevant allergen extracts of regional importance. Pollen is a vital aeroallergen, and there were reports on the association of pollen sensitization with allergic rhinitis in Taiwan and Korea (38, 39). However, little is known about pollen allergy and pollinosis in tropical Asia, especially in Vietnam. Thus, further studies are necessary.

In conclusion, this study reinforced that mites and cockroach are most common aeroallergens in patients from the south of Vietnam, with a slight difference in patterns of mite species depending on the geographical regions of Vietnam. Sensitizations to Df and Dp were associated with male gender and younger age, respectively. Sensitizations to both HDM and storage mites were associated with urticaria, AD and AR. Polysensitization to HDM and storage mites was common among the Vietnamese population. Taken together, the results from this study can create a platform for further studies focusing on allergic diseases in southern Vietnam.

## Data Availability

The original contributions presented in the study are included in the article/Supplementary Materials, further inquiries can be directed to the corresponding author/s.
